# Supplementary material for: Risk Factors and Spatiotemporal Analysis of Classical Swine Fever in Ecuador
Source: Viruses. 2023 Jan 19;15(2):288. doi: 10.3390/v15020288 (PMC9966056; doi:10.3390/v15020288)
Supplement: Supplementary file 1 [file viruses-15-00288-s001.zip › suplementary/SM1-3.pdf]

Risk factors and spatiotemporal analysis of classical swine fever in Ecuador

Alfredo Acosta*[1,2], Klaas Dietze[1], Oswaldo Baquero[2], Germana Vizzotto Osowski[2], Christian Imbacuan[3] , Lidia Alexandra Burbano[3], Fernando Ferreira[2,], Klaus Depner*[1]

[1] Institute of International animal health/One health, Friedrich-Loeffler-Institut, Greifswald, Germany.
[2] Laboratory of epidemiology and biostatistics, School of Veterinary Medicine and Animal Science, Preventive Veterinary Medicine Department, University of São Paulo, Sao Paulo, Brazil.
[3] General coordination of animal health, Phyto-zoosanitary Regulation and Control Agency, Quito, Ecuador.

* Correspondence: Alfredo Acosta alfredoacosta@alumni.usp.br, Klaus Depner Klaus.Depner@fli.de

Supplementary materials

SM1 Questionnaire

-Premises general information

The introduction section of the questionnaire includes General questions (n=11) about the premise location, such as name of the province, canton, parish, and geographic coordinates (UTM); Farmer demographics questions (n=8), names of the farmer, contact information, national identification number and type of production (backyard, familiar, commercial and industrial); Chronology questions (n=7) included date of the visit, date of first clinical signs that motivated the suspicious, the person who did the notification, unique ID of the event (generated by the system), species of animals affected and the pathology noticed by the person who did the notification.

-Clinical signs of pigs and sampling

This section contains questions (n=12) about health status, symptomatology, sanitary practices on the farm, details about the first clinical signs of suspicious animals, and feed characteristics. The samples section includes questions (n=12) about sample characteristics, quantity, date of collection, shipment, requested test, laboratory and results.

-Vaccination information

The vaccination section includes questions (n=6) about immunisation against CSF. We checked the official campaign vaccination records, considering a vaccinated premise if it was vaccinated 6 months before the day of onset of clinical signs (mandatory vaccination interval).

-Herd population and movements

Pig population questions (n=12) included the number of susceptible, sick, dead, and culled pigs. Movement questions (n=8) included information on pig arrivals or departures in the past 30 days.

SM2 Logistic regression model

The variables were inserted following a stepwise forward model construction (table 1), no evidence of collinearity (table 2).

Table S1. Multiple regression model construction

Variable	Category	M1	M2	M3	M4	M5	M6
Intercept							
Swill feeding	No	1	1	1	1	1	1
	Yes	7.12 (4.16–13.29)	8.09 (4.69–15.20)	8.05 (4.66–15.17)	8.53 (4.92–16.11)	9.34 (5.35–17.74)	9.25 (5.31–17.53)
Time until notification	1–7 days		1	1	1	1	1
	>7 days		2.66 (2.02–3.50)	2.50 (1.90–3.30)	2.44 (1.84–3.23)	2.22 (1.67–2.95)	2.20 (1.43–3.37)
Introduction of new pigs (last 30 days)	No			1	1	1	1
	Yes			2.07 (1.56–2.70)	2.01 (1.51–2.67)	2.07 (1.55–2.77)	2.14 (1.59–2.86)
Vaccination record CSF	Yes				1	1	1
	No				1.82 (1.39–2.38)	1.86 (1.42–2.45)	1.82 (1.38–2.39)
Natural Region	Highlands					1	1

	Coastal					1.88 (1.35–2.61)	2.34 (1.55–3.55)
	Amazon					0.89 (0.64–1.23)	0.72 (0.47–1.08)
Region : Notification	>7 days:Coastal						0.61 (0.31–1.17)
	>7 days:Amazonic						1.95 (0.96–3.99)
Chi-sqrt		2.2 exp-16	2.79 exp-12	6.12 exp-7	1.23 exp-05	7.05 exp-05	0.011
Signif		***	***	***	***	***	*
AIC		1392	1345	1323	1305	1290	1286

Table S2. Collinearity Variance inflation factors results

**Variables**	**GVIF**	**Df**	**GVIF^(1/(2*Df))**
Swill feeding	1.024	1	1.012
Time until notification	1.028	1	1.014
Introduction of new pigs	1.021	1	1.010
Vaccination record CSF	1.012	1	1.006

We observed adequate distributions of residuals, indicating a good adjustment of the model. There were any outliers with a significant influence on model fitting, according to Bonferonni outlier test (p=0.008). However, there were influential observations, such as case numbers 27 and 426, according to Cook’s distance (Figure 1).

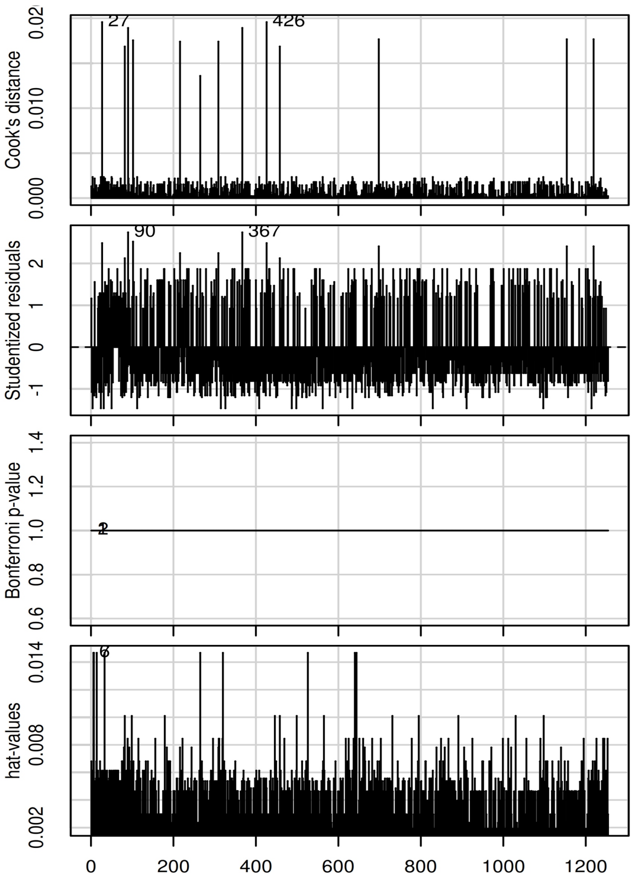

Figure S1. Residuals analysis and influence index

The spatial predicted probability of the logistic model used parish information; there was epidemiological silence in 599 parishes throughout the study period (Figure 2). The receiver operator curve showed an AUC of 0.72 (Figure 3). Predicted probability on Fig. 4.

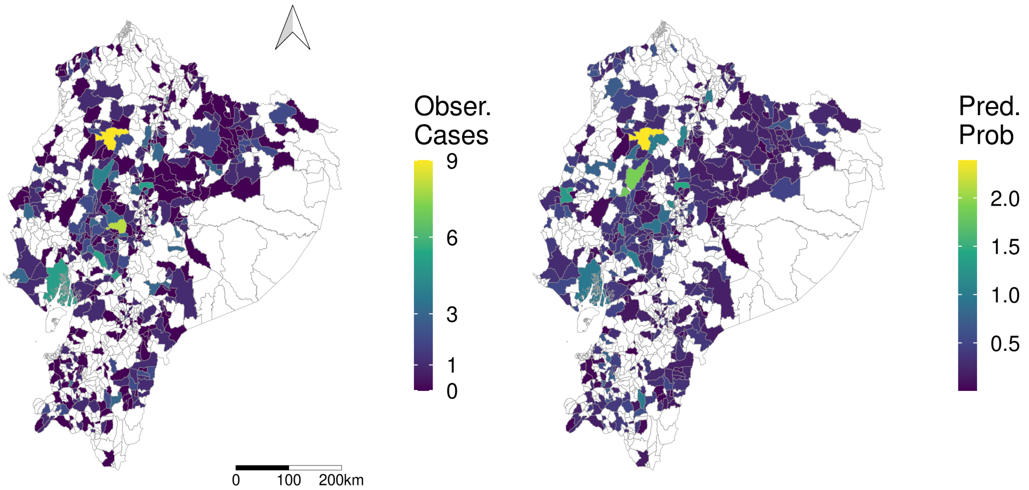

Figure S2. Spatial distribution of observed cases (left) and the prediction values (right) of the multivariable lo-gistic model, aggregated by parishes between 2014 and 2020. Parishes without information of cases or con-trols are white.

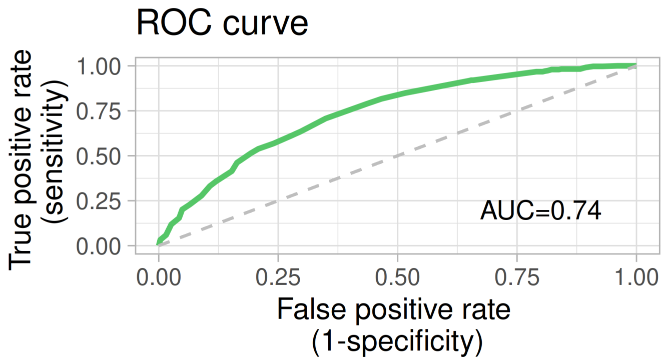

Figure S3. Receiver operator curve.

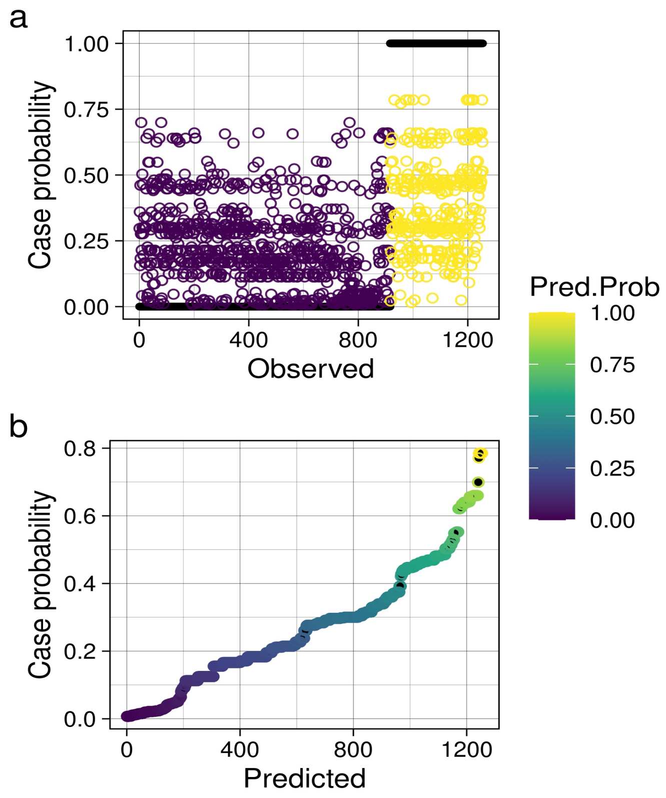

Figure S4. Predicted probability of the ultimate model a) Observed probability, b)Predicted probability.

SM 3 Bayesian model

The expected numbers of cases were 34, 40, 38 and 32 from 2017 to 2020, respectively. They were most widely distributed in the central and northern highlands (Guaranda, Latacunga), and in the central-western area of high pig production (Santo Domingo-El Carmen) (Fig. 5).

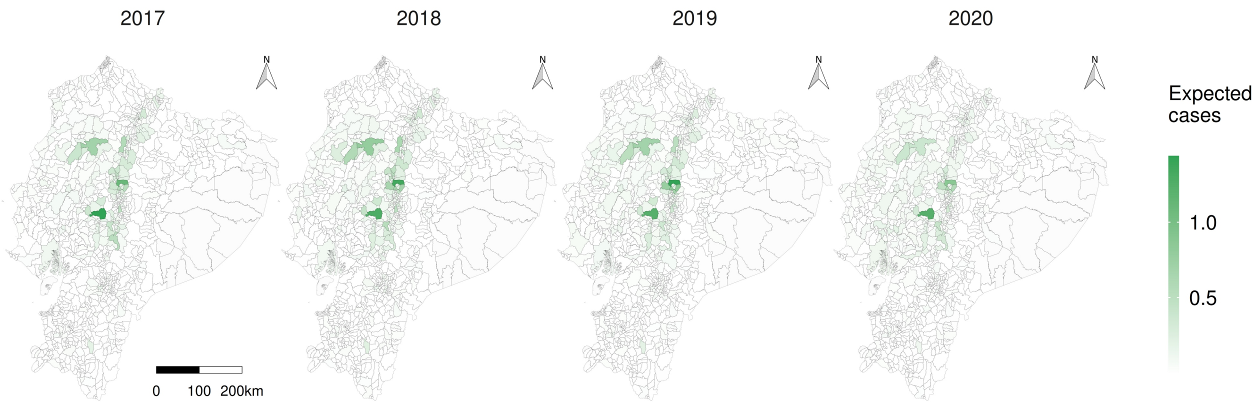

Figure S5. Representation of expected cases in Ecuador from 2017 to 2020, considering higher density population expected were higher in central highland zone and north wester in *Santo Domingo*.

Vaccination coverage is heterogeneous and well dispersed all over the country available in (Fig. 6). It is possible to visualise higher coverage in the western centre (Santo Domingo-El Carmen), the northern (Carchi) and western southern (El Oro) were highly influenced by industrial premises. In the central Andean (Cotopaxi, Chimborazo) associated with backyard production. There were 110, 84, 60 and 69 parishes without doses applied in 2017, 2018, 2019 and 2020, respectively.

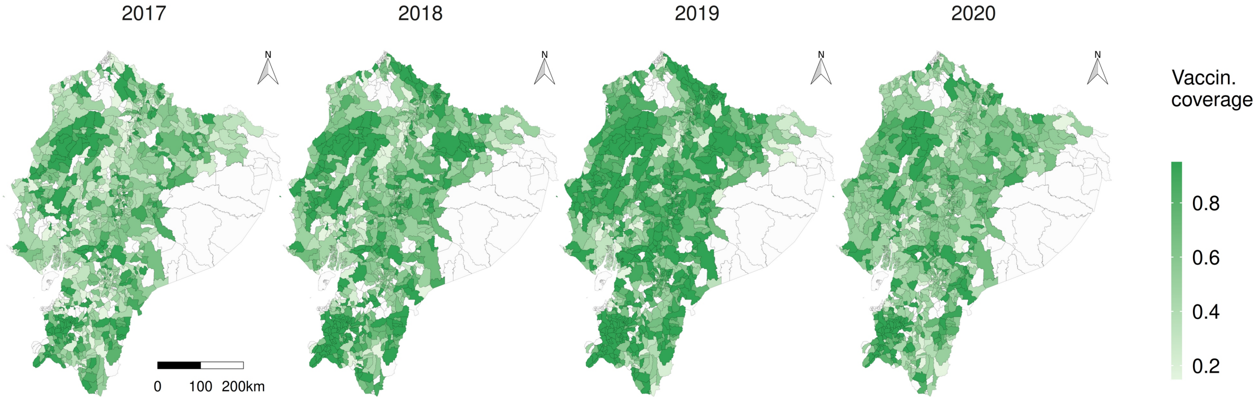

Figure S6. Map of parish level vaccination coverage in Ecuador.

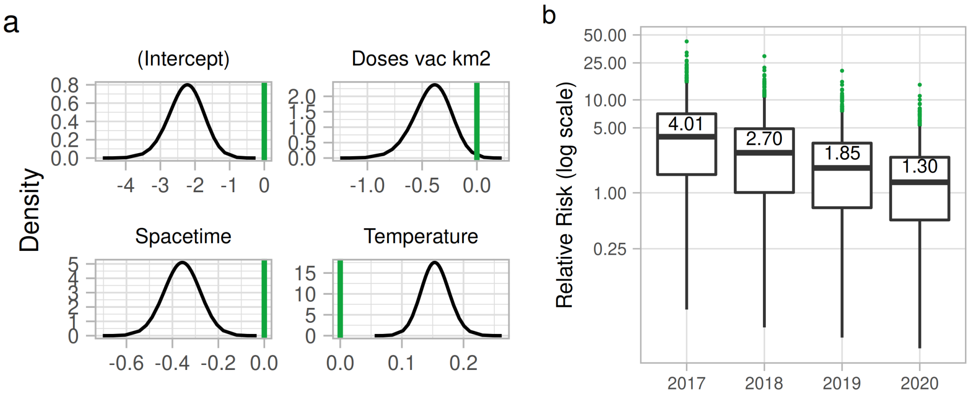

Figure S7. a) Spatiotemporal model posterior distributions (significant covariates on green). b) Boxplot of aver-age parish relative risk (RR) by year (median values shown on the boxplots).

Table S3. Average parish relative risk of Classical swine fever in the 23 provinces of Ecuador (2020), showing possible prioritisation province order of governmental actions to reduce the risk.

Province code	Name of province	Average Relative Risk
14	Morona Santiago	3.68
12	Los Rios	3.12
24	Santa Elena	3.07
16	Pastaza	2.86
08	Esmeraldas	2.83
09	Guayas	2.82
19	Zamora Chinchipe	2.81
07	El Oro	2.65
22	Orellana	2.44
11	Loja	2.19
90	Manabi	1.83
21	Sucumbios	1.5
02	Bolivar	1.2
15	Napo	1.18
03	Cañar	1.09
01	Azuay	0.98
23	Santo Domingo	0.93
04	Chimborazo	0.55
18	Tungurahua	0.55
05	Cotopaxi	0.53
17	Pichincha	0.47
90	Imbabura	0.41
04	Carchi	0.38
